# Defense against small parties: electoral reforms and their impact on Tunisia’s electoral system since the Arab Spring

**DOI:** 10.1007/s12286-023-00561-z

**Published:** 2023-03-08

**Authors:** Hager Ali

**Affiliations:** GIGA Institute for Middle East Studies, German Institute for Global and Area Studies, Neuer Jungfernstieg 21, 20354 Hamburg, Germany

**Keywords:** Electoral Reforms, Electoral Legislation, Tunisia, Electoral Competition, Representation, Reformen, Wahlrecht, Tunesien, Wahlwettbewerb, Repräsentation

## Abstract

Tunisia’s political system suffers from recurrent problems with governability and proportionality. A volatile party landscape, frequent cabinet reshuffles, and political gridlocks repeatedly jeopardized stability and democratic progress since the Arab Spring. Major and minor electoral reforms were undertaken in 2014, 2017, 2019, and 2022, but they were unable to diffuse either of these issues. This analysis is therefore driven by two central questions: How have Tunisia’s electoral laws changed since the Arab Spring? And why have these reforms failed to improve both governability and proportionality? It will be argued that because Tunisia’s party landscape is fractured and volatile only on the secularist side of the spectrum, coalitions with and against Ennahda are costly to all parties involved, worsening the overall quality of political representation. By analyzing the trajectory of major and minor electoral reforms longitudinally, this paper finds that Tunisia’s electoral reforms incrementally restricted the electoral system by limiting parties’ and candidates’ capacity to compete in elections. Through modifying legislation on campaign finance and subsidies, gender parity, and candidacy requirements, and finally abolishing Tunisia’s closed list PR-system in 2022, reforms benefit established older parties and wealthier candidates while fortifying the electoral system against newer and less wealthy contenders. Tunisia’s electoral reforms are inadequate in addressing governability and proportionality because restricting electoral competition alone cannot improve the quality of representation through political parties.

## Abstract (de)

Seit dem Arabischen Frühling wird Tunesiens demokratische Stabilität durch häufige Kabinettsumbildungen, politische Stagnation, und einer volatilen Parteienlandschaft gefährdet. Regierungsfähigkeit und Proportionalität sind wiederkehrende Herausforderungen geblieben. Obwohl in den Jahren 2014, 2017, 2019, und 2022 Wahlrechtsreformen durchgeführt wurden, konnten diese Probleme jedoch nicht tiefergehend behoben werden. Diese Analyse verfolgt daher zwei Leitfragen: Wie hat sich Tunesiens Wahlgesetzgebung seit 2011 verändert? Und warum konnten diese Reformen weder Regierungsfähigkeit noch Proportionalität in der politischen Repräsentation verbessern? Da die tunesische Parteienlandschaft hauptsächlich auf der säkularen Seite fragmentiert und volatil ist, werden Koalitionen sowohl mit als auch gegen die konservative Ennahda-Partei kostspielig für alle Beteiligten. Das vermindert die Repräsentationsfähigkeit vieler Parteien und begünstigt politische Gridlocks. Die Analyse des tunesischen Wahlrechts und dessen Verlauf zeigt auf, dass der Zugang zu politischem Wettbewerb zunehmend restriktiver wurde. Änderungen in der Gesetzgebung zur Finanzierung von Kampagnen, Genderparität, und den Voraussetzungen für Kandidaturen bringen Wettbewerbsvorteile für etabliertere und wohlhabendere Kandidaten und Parteien. Neuere Wettbewerber mit weniger finanziellen Ressourcen werden hingegen strukturell benachteiligt und so vom Wettbewerb ausgeschlossen. Tunesiens Wahlgesetzgebung trug daher wenig zur Verbesserung von Proportionalität und Regierungsfähigkeit bei, da eine restriktivere Wahlarena die durch intra-parteiliche Zersplitterung verminderte Repräsentationsfähigheit von Parteien nicht beheben kann.

## Introduction

Since former president Ben Ali was ousted in the Arab Spring in 2010, Tunisia’s path to democratic stability was turbulent at best. In the country’s most recent political crisis in July 2021, the Covid-19 pandemic brought the Tunisian healthcare system to the brink of collapse, which fuelled mass protests calling for major political change (Amara 2021; Reuters 2021). To regain control of the situation, President Kais Saied froze the National Constituent Assembly, and sacked prime minister and independent political rival Hichem Mechichi, who had strong backing from the Ennahda party (Jawa 2021).

Observers flagged Saied’s actions as an undemocratic takeover or self-coup (Sarah and Al-Mailam 2022). Changes introduced to the political system chipped away at democratic progress under the guise of re-stabilizing the country (AFP 2021). A constitutional referendum in July 2022 established a bicameral parliament and Tunisia’s semi-presidential political system became a presidential system (France24 2022). A Presidential decree in September 2022 (Presidential Decree Law No. 2022-55 2022) then introduced major electoral reforms that transformed Tunisia’s electoral system from a closed-list PR-system to single-member districts which sideline political parties, though the electoral process for presidential elections remained unchanged at the time of writing (Aliriza 2022).

Even before the 2021-crisis, widespread protests were a recurring phenomenon, as were the calls for electoral reform which became ubiquitous at least since the 2014 constitution was ratified (Labidi 2019). Prior to the 2022-decree, one major electoral reform was undertaken in Tunisia through the 2014 electoral law (*Loi organique n° 2014‑16 du 26 mai* 2014), with smaller amendments in 2017 and 2019. Yet, frequent cabinet reshuffles and political gridlocks remained characteristic for Tunisia’s political landscape (Yerkes and Ben Yahmed 2019). That indicates a deficient capacity for proportional representation, which is rather surprising in what used to be a PR-system.

Four electoral reforms within less than a decade failed to stabilize a new and fragile democracy. That should warrant a closer look into the intricacies of electoral law and its reforms. In the forerun of elections, the electoral laws governing the rules of the electoral game can become contentious and in need of reform, especially when the political landscape changes as drastically as it did in Tunisia since 2011. When electoral reforms fail to mitigate major problems with governability within cabinets or proportionality in political representation, younger or less consolidated democracies become vulnerable to gridlocks, protracted crises, and takeovers. Electoral reforms can indeed improve proportionality and representation in a given political system. But to varying extents, electoral reforms can also be used to fortify the political arena from specific contenders in any regime, whether democracies, hybrid regimes, or autocracies.

Minor electoral reforms are particularly overlooked in the existing literature (McElwain 2008), and even fewer longitudinal case studies dissect the full trajectory of electoral legislation in a regime. Changes to electoral systems are not dichotomous, but rather a gradual process wherein smaller segments of electoral legislation can be adjusted between elections depending on the political landscape, partisan interests, and an incumbent’s political goals (Rahat 2014). When new regimes emerge after incisive political events, such as regime breakdowns or democratization processes, the first generation of relevant political actors can imprint long-term interests into the policy-set up of an electoral system as no electoral system is completely neutral in how it transposes votes into seats (Patkós and Stump 2022).

This paper will compare and analyze electoral legislation in Tunisia since 2011 to investigate two central questions: How have Tunisia’s electoral laws changed over time? And why have these reforms consistently failed to improve governability and proportionality? It will be argued that, although there typically is a trade-off between governability and proportionality, Tunisia’s electoral system is lacking in both. The party landscape is highly volatile and fractured, though factionalism is more due to intra-party politics than the representation of niche interests. As parties frequently split and merged on the secularist side of Tunisia’s political spectrum, governance without the conservative Ennahda party was difficult and costly to all parties involved. At the same time forming electoral alliances against Ennahda forced many parties into short-lived alliances and centrist positions, which in turn worsened the overall quality of political representation.

The analysis finds that Tunisia’s electoral reforms focus on issues pertaining to campaigning logistics, finance, candidacy requirements, suffrage of security forces and women, which determine a new political contender’s chances to enter the political arena in electoral competition. When taken together, Tunisia’s electoral reforms incrementally limited access to the electoral playing field by raising the administrative and financial obstacles of contenders. The formation and dissolution of new parties as well as coalition-building in the forerun of elections has, however, been consistently overlooked in reforms despite the dominant role of party politics in each of Tunisia’s major political crises since 2011. The 2022 reform then levered out the role of political parties altogether, because new districting and abolishing the previous closed-list PR system emphasizes the role of specific candidates over parties. At the same time, new candidate requirements and campaign finance criteria tilt the electoral competition in favour of wealthier candidates.

Each major and minor reform before 2022 failed to improve governability and proportionality because Tunisia’s electoral laws skirt issues of party formation, membership, and coalition-building during electoral campaigns altogether. Wealthier candidates and established parties prior to the 2022-reform gained a structural advantage through these reforms, while the electoral system became less inclusive towards smaller, less wealthy, and new political contenders in electoral competition. The structural bias towards wealthy contenders became more pronounced through the 2022 reform, which abolished state-subsidies for campaign finance entirely. Access to electoral competition is conditional on a candidate’s personal assets while smaller single-candidate districts give a new edge to existing intra-party divisions.

The case of Tunisia is particularly suitable to shed light on the trajectory of reforms for several reasons. Of course, given recent developments in Tunisia and having both minor and major electoral reforms within less than a decade, this case study could not be timelier. Electoral reforms and their negotiation by the first generation of political actors in a new polity can potentially make or break transitional processes. Showcasing the trajectory of electoral legislation with its most likely benefactors parses the fine-grained legal prerequisites of regime change, whether the transition is towards democracy or away from it. The timeframe of this analysis on the other hand covers electoral reforms through Tunisia’s democratization and its recent backsliding process.

This paper is structured into six sections and a conclusion. Sections two and three review relevant literature on electoral reforms, and outline the basic properties of Tunisia’s party landscape as an important reform-driver. The analysis in section four and five will first lay out major and minor electoral reforms since the Arab Spring by comparing the electoral laws of 2014, 2017, 2019, and 2022 (see Table 1). The 2014 electoral legislation is covered in more detail as the legal point of reference to all subsequent reforms. Because the original laws were not available in English, the electoral laws from 2014, 2017, and 2019 were translated from French, and the 2022-reform from Arabic by the author. Lastly, section six and the conclusion will discuss how these reforms address proportionality and governability in correspondence to the second research question, and important takeaways from this analysis.

**Table 1:** Overview of notable electoral reforms 2014, 2017, and 2022 and how they impact electoral competition, sorted by reform issue. Table compiled by author from electoral laws of 2014, 2017, and 2022

Reform issue & year		Notable electoral legislation	Impact on elec. competition
*Electoral system*	*2014*	**Art. 7**: Establishes closed-list PR-system. Registration for elections is voluntary. **Art. 10:** Political parties and blocs submit candidate lists	(Creates path dependency of electoral system and main basis for all subsequent amendments)
	*2017*	**Art. 7:** Registry for elections becomes mandatory and personal	Likely to increase voter turnout
	*2022*	**Annex A–B**: Size of districts was decreased, number of total districts increased. One candidate per district is elected in two rounds	Prioritizes candidates over parties, smaller districts emphasize local/regional affiliations and issues in campaigning over party affiliation
*Suffrage*	*2014*	**Art. 6: **Citizens detained for mental incapacitation, military and security personnel prohibited from registering to vote	Restrictions on suffrage for security and military personnel are in line with the 2014 constitutional provision on military neutrality
	*2017*	**Art. 6, 49, 52: **Restrictions on military and security personnel lifted only for municipal and regional elections, but soldiers and security personnel may not run as candidates, participate in campaigns, partisan meetings, or any other election-related activities. Soldiers and security personnel violating these provisions will be dismissed	Military and police are historically secularist because coercive apparatus was used to keep Islamists in check. Potential competitive advantage for secularist actors offset by general conscription, however
	*2022*	**Art 6:** Voter registration excludes active-duty military and security personnel again and was extended to anyone detained for any reason	Vague language on reasons for detainment facilitates exclusion of specific voters and candidates
*Candidacy*	*2014*	**Art. 19, 21: **Parties and lists apply on behalf of all candidates by submitting names, copies of IDs, and tax declarations to ISIE. Any Tunisian over 23 years and anyone who acquired Tunisian citizenship at least six years prior can run as candidate	Shift from parties/lists to individual applications with signatories increases administrative burden on candidates wishing to enter electoral competition. New legislation effectively excludes dual citizens and restricts naturalized citizens. Gender and age requirements of signatories and their exclusivity fuels competition between specific candidates at the expense of party cohesion
	*2022*	**Art. 19–21: **Individuals apply for candidacy. Candidates must be Tunisian, or born to a Tunisian parent, not have any other citizenship, be 23 years old, must not have been charged with any crime, and must reside in the constituency they are running in. Members of government and heads of government offices, Imams, and leaders of sports associations are not eligible for candidacy for a year after serving in office. Candidates need to submit campaign plans in addition to 400 signatures from voters in their constituency, who may not endorse any other candidate. Half of these signatures must be from women, and a quarter of them from voters under 35 years of age. **Art. 161**: Exploiting another candidate’s honor, regional, local, or familial affiliations is punishable by imprisonment for two to five years and canceling the candidate’s votes	
*Parity/inclusion*	*2014*	**Art. 24–25:** Parity between women and men in candidacies with alternation between female and male candidates in lists is required. Candidates younger than 35 years must be included among top four on lists in constituencies with at least two seats. Lists will lose half their subsidies if criteria are not met	Competitive advantage depends on location and social class. If urban middle-class women with higher education, state-driven feminism benefits secular parties. If rural lower and lower middle class, parity caters to conservative parties
	*2017*	**Art. 49:** Gender parity extended to municipal and regional elections, also applies to heads of lists and coalitions. One candidate with a physical disability must be included among top ten candidates	
	*2022*	(Parity criteria not applicable anymore due to electoral system change)	–
*Campaign period*	*2014*	**Art. 50:** Election or referendum campaigns begin 22 days before voting. Pre-electoral or pre-referendum phase is set for three months	Short campaign period advantages older and more institutionalized parties who do not need to build a new voter base through campaigning
	*2017*	**Art. 50:** Duration of election or referendum campaigns remains 22 days before voting, but pre-electoral and pre-referendum phase reduced to two months	
*Campaign finance/subsidies*	*2014*	**Art. 77–78, 80:** Campaign finance in cash or in-kind through natural persons only. Contributions from foreign sources, corporations, governments, or foreign individuals are prohibited. Every candidate/list may receive public subsidies for campaigning. First half to be paid before campaigning, second half a week after final electoral results. If candidates or lists achieve below 3% of votes or fail to gain seat in legislature, public subsidies must be paid back	Restrictions on funding sources and subsidies advantages wealthier candidates, lists, or parties because they require sufficient financial resources to compete in elections. Members of former elites generally wealthier, while younger citizens afflicted by socio-economic grievances likely cannot afford to run for office. Restrictions on foreign funding can be instrumentalized against specific candidates and parties
	*2017*	**Art. 2, 78:** Modification of payment of subsidies. Public subsidies now paid as lump-sum reimbursement after final electoral results, provided that financial statements were filed with the court of auditors. Public funding cannot exceed self-financing of a candidate or list. Expenditure limits are subject to size of electoral districts and electorate, living expenses, and decrees. Candidates who do not publish financial statements, will not receive subsidies for electoral expenses	
	*2022*	**Art. 75, 81:** Electoral and referendum campaigns are to be exclusively financed through self- and private funding. The upper limit for spending on electoral or referendum campaigns is determined by an order after consulting the commission	Additional financial restrictions on candidates make electoral competition less inclusive for candidates from poorer socio-economic backgrounds
*Funding penalties*	*2014*	**Art. 98:** Introduces penalties in case of failure to provide financial statements and electoral spending. If Court of Auditors rejects financial statement, the penalty is dependent on the amount exceeding financial limits	Penalties raise stakes on infractions concerning campaign finance
	*2017*	**Art. 98: **Penalties dependent on maximum amount of public subsidy in each constituency, rather than excess expenditure, if financial statements are rejected by the Court of Auditors. If surplus of spending exceeds 75%, financial penalty will be five times the excess amount, plus forfeiture of candidate’s mandates	
	*2022*	**Art. 161, 163:** Candidates and legislators shall be punished with imprisonment and a fine between 2000 and 5000 Tunisian Dinars, if they used monetary or in-kind donations to influence voters before or after polling. If found guilty, candidate loses the right to candidacy for life and the right to vote for 10 years from the date of conviction. No candidate, list, or party may receive anonymous or foreign campaign funding. If anonymous or foreign funding is accepted, the penalty will be a fine between 10 and 15 times of the received funding, five years of imprisonment, and a ban from running in any elections after the conviction	New penalties can effectively eliminate candidates from any and all elections

## Leveraging electoral reforms against new contenders

Survival in political office by virtue necessitates securing votes and seats. Electoral systems shape political parties, party systems, how politicians behave and how voters strategize when electing legislators (Mainwaring 1991). It is difficult to study the origins of electoral systems rather than their consequences because there are not enough cases to be studied quantitatively (Shugart 2005). Previous literature mostly dealt with the political consequences of electoral laws through large‑N analyses or through case studies about one particular reform (most notably Lijphart 1990).

The rules that make up an electoral system regulate electoral competition, campaign logistics, coalition building, candidate requirements, finance, and how votes are transposed into seats. No electoral system however transposes votes in a completely neutral manner—every type of electoral system may structurally benefit some political actors over overs (Patkós and Stump 2022). Political actors may be motivated to alter electoral systems by partisan concerns, pursuit of power and influence, seats,, their respective values, or the timeframe of their goals (Renwick 2010). The prevailing party system often also determines electoral system choices (Colomer 2004). Conversely, when incumbents already benefit from the system in place, or make miscalculations about their own power and vulnerability, electoral systems may not be changed and fortified instead (Colomer 2004, pp. 61–62). That makes the design of electoral reforms very tricky (Sartori 1994). The initial choice of electoral systems after critical events—including civil wars, gaining independence, and regime breakdowns—is typically subject to the strategic calculations of the main political actors at that point in time (McElwain 2008, p. 33).

Though “macro-level” changes in electoral systems concerning district magnitudes and electoral formulas are rare (Katz 2005), “micro-level” features can be altered much more frequently (McElwain 2008, p. 33). Unfortunately, existing research underestimates how often electoral reforms have taken place precisely because such ‘micro-reforms’ are overlooked (McElwain 2008, p. 33). Another reason is the treatment of political parties as cohesive units; intra-party factionalism and rivalry between party leaders and party members can be potent drivers of reforms (McElwain 2008, p. 35). Regulations concerning campaigning and campaign finance, such as caps on spending, fundraising or disclosure, are common targets of smaller reforms—or manipulation—in electoral legislation (Birch 2011, p. 30). Consequently, electoral reforms are not a dichotomous outcome, but rather gradual processes (Rahat 2014, p. 539). Change is adopted incrementally. Across non-democratic polities, such as electoral autocracies, incumbents frequently meddle in electoral legislation to disarm their opposition through gradual changes and adjustments (Schedler 2006).

When electoral results are consistent and predictable, retaining the existing set of electoral rules is usually advantageous to incumbents (Remmer 2008). Extreme electoral volatility, however, can drive politicians to change electoral legislation in hopes to counterbalance voters’ preferences (Núñez et al. 2017). In this context, electoral systems may be fortified against electoral newcomers to preserve the electoral playing field and prevent fragmentation, especially when new parties could disturb coalition bargaining and government stability (Núñez et al. 2017, p. 379). When new parties pose a serious threat to incumbents, they are also found to drive reforms that make electoral systems less inclusive to contain challengers (Núñez et al. 2017, p. 394). A change in the salience of cleavages can provoke adjustments through reforms (Hamid 2014).

There is consensus that larger parties prefer single-member plurality (SMP) systems because they can win disproportionately more seats under SMP, whereas smaller parties tend towards PR-systems (McElwain 2008, p. 32). Single non-transferable vote (SNTV) systems on the other hand are rarely used. As SNTV systems prompt voting for specific candidates, they can uproot political parties especially where tribal or ethnoreligious identities play a dominant role in politics (Hamid 2014, p. 136).

The classic literature on electoral reforms is heavily Eurocentric, though newer research is gradually shifting its focus to electoral reforms in global south polities. Research on political change in the Middle East and North Africa (MENA) also focused more on electoral or policy outcomes, whereas the specific policies towards those outcomes remain comparatively understudied. The Arab Spring provoked several major and minor reforms which either aimed to preserve the political status quo, respond to acute challenges, or reverse tendencies of the preceding political system. Jordan’s electoral system prior to the Arab Spring used an SNTV system and adopted an open-list PR-system in 2011 to foster more electoral alliances among candidates (Carey and Reynolds 2011, pp. 41–43). When a vocal opposition block emerged in Kuwait’s 2012 election, on the other hand, the Emir introduced a SNTV-system to specifically undermine electoral coalitions and prevent similarly strong opposition alliances (Tavana 2018). When the military upended Sudan’s 2019 democratization process through a coup, it introduced reforms with the purpose of gaining unfettered access to the electoral commission ahead of the planned 2023 elections (Ali et al. 2022). In Libya’s 2012 elections to the General National Congress, a SNTV-system was adopted precisely because it favours independent candidates, which would foreseeably prevent the hegemony of specific political parties while working towards its first constitution since Gaddafi’s rule (Hamid 2014, p. 137). Though this is only anecdotal, the instrumentalization of electoral legislation against political parties across the MENA-region is striking, whether to uphold regimes or to change them. Saied may not have imposed a SNTV-system in Tunisia, but the drastic change towards single-member districts in 2022 similarly neutralises political parties.

## Tunisia’s fragmented party landscape as driver and addressee of reforms

Related to the previous critique that existing research often disregards minor electoral reforms is that reforms are often studied in isolation from their key drivers, such as party system fragmentation. Tunisia’s highly volatile and fragmented party landscape in conjunction with its cleavage structures repeatedly drove calls for reform and popular mobilization (Gobe 2017). Tunisia’s party system does not neatly fit into the classic structured-versus-unstructured classification of party systems (Sartori 1994). The conservative catch-all party Ennahda has a long history as well as established financial and human resources and is comparatively well-institutionalized. At the other end of the spectrum are secularist and liberal parties predominantly founded after 2010. Splits, merges, and short-lived alliances disrupted continuity in legislature and cabinet. In total, there have been ten cabinet reshuffles since 2011, and most parties failed to establish clear platforms, organizational infrastructure, and to build partisanship (Yerkes and Ben Yahmed 2019).

Between colonial independence and the coup of 1987, Bouguiba’s secular *Parti Socialiste Destourien* (PSD) had been dominant in the Tunisian party landscape (Boulby 1988, p. 590). Prompted by reforms after colonial independence and the dismantlement of Islamic institutions by modernizing elites, an Islamist countermovement emerged in the 1970s (Boulby 1988, p. 591). Between 1967 and 1973, Tunisia faced an economic crisis largely because of failed socialist reforms and socialist planning (Boulby 1988, p. 597). The main counterweight to the PSD was the Tunisian radical pan-Islamist *Hizb ut-Tahrir* party, the Islamic Tendency Movement—Ennahda’s predecessor—as well as student movements and communist parties. After his coup against Bourguiba, Ben Ali renamed the PSD into the Democratic Constitutional Rally (RCD) which then held sole power over Tunisia until it was dissolved in 2011.

Ahead of Tunisia’s 2013 crisis, a Troika consisting of the Ennahda party, *Congrès pour la république* (CPR), and Ettakatol versus the newly formed Nidaa Tounes was in charge (Boubekeur 2015, p. 114). Nidaa Tounes was founded in 2012 as a catch-all movement against the Troika, and later allied with the Republican Party, Socialist Party, Patriotic and Democratic Party, and al-Massar (Boubekeur 2015, p. 117). The Troika proved to be highly dysfunctional as it was unable to put forward any strategy to tackle Tunisia’s economic, security and political crisis (Boubekeur 2015, p. 114). Both Ennahda and Nidaa Tounes leveraged the street protests and general ideological polarization to fasten their grip on institutions, rather than providing a political arena to resolve differences (Boubekeur 2015, p. 117). The 2014 election then proved to be a critical juncture for Nidaa Tounes. Initially, electoral alliances between upper and middle classes from coastal regions, secular leftists, civil servants, former regime members, and businesspeople have helped assert Nidaa Tounes as a counterweight to Ennahda, but its internal heterogeneity hindered consensus about future institutions or reforms (Boubekeur 2015, p. 121). As a result, party members and voters frequently migrated towards other secular parties or founded new ones.

Ennahda was able to retain its electoral base through mobilizing disciplined voters and welfare activism (Sadiki 2011). Most secular political parties in Tunisia’s parliament on the other hand were founded after 2011. Afek Tounes Party for example was founded in 2012 but merged with other secular parties to form the Republican Party, which then disappeared after 2019. A significant portion of seats in the parliament was also held by independent candidates (Yerkes 2019).

Amid six cabinet reshuffles between 2014 and 2019, at least eight new secular parties were newly founded, or split off from Nidaa Tounes (Yerkes and Ben Yahmed 2019). Many of them failed to develop distinct party platforms, often relying on the identity of the party founder or leader (Yerkes and Ben Yahmed 2019, p. 6). Forced consensus-building between secularists and Islamists, in part through Rapprochement-politics and the Carthage-Agreement in 2016, hindered forming distinct party identities and building stable voter bases (Dihstelhoff and Sold 2016, p. 2). The high frequency of cabinet reshuffles, fragmentation of the party landscape in addition to parties’ disconnect from voters, set the stage for major electoral reforms with smaller adjustments between 2014 and 2022.

## Tunisia’s major and minor electoral reforms from 2014 to 2019

Until the 2022 constitutional referendum, Tunisia’s national legislature was unicameral (Constitution of 2014, Art. 50). In 2011, the electoral commission redesigned Tunisia’s new electoral system. Tunisia’s electoral system became a closed list PR-system where individual seats were distributed using the largest remainder method (Battera and Giuseppe 2019). Elections and referenda are organized and managed by the Supreme Independent Elections Commission (ISIE) (Constitution of 2014, Art. 126), whose budget is administered by the legislative assembly. Electoral law is regulated through organic legislation adopted by absolute majority (Art. 64).

The Electoral law of 2014 (*Loi organique n° 2014-16 du 26 mai* 2014)^1^ was the first major reform after the Arab Spring, providing the basis for subsequent minor reforms in 2017 and 2019, and the most recent major reform in 2022, which are summarized in Table 1. It is divided into seven chapters with general provisions concerning its promulgation, provisions concerning voters, candidates, the electoral and referendum period, ballots, scoring, the announcement of electoral results, electoral infractions, and transitory provisions. Citizens need to actively register in person to vote, though voter registration itself is voluntary (Art. 5, Art. 7–8). Military and internal security forces are explicitly not allowed to register as voters (Art. 6). Municipal administrations need to submit data on persons prohibited from voting in certain intervals (Art. 9). The duration of electoral campaigns is also limited; electoral and referendum campaigns open 22 days ahead of polling, close 24 h ahead of polling, and are preceded by a pre-electoral or pre-referendum phase which is three months long (Art. 50). During campaigns, places of worship and national media are to be neutral. Funding sources of campaigns and spending must be transparent (Art. 52) and public resources cannot be used for candidates or parties (Art. 53).

Article 57 prohibits political advertising “in all cases during the electoral period”. Partisan newspapers may advertise during the electoral campaign for their respective party, candidate lists, and specific candidates only. Announcements through media are prohibited during the election period, as is the establishment of free phone lines, mailboxes, or call centres in favour of a candidate, list, or party (Art. 58). The means for electoral and referendum campaigns are announcements, public meetings, demonstrations, processions, rallies, as well as some audio-visual media and electronic media (Art. 59). Announcements concerning elections and referenda consist of posters, leaflets, programmes, and announcements of meeting dates (Art. 60). Municipalities determine the specific locations for electoral posters and the allocation of poster spaces for each candidate or party (Art. 62). Parties or candidates are also not allowed to give away their reserved poster-spaces or use the spaces for other purposes (Art. 63).

There is also an electoral silence on election day until polling stations close, where campaigning, opinion polls directly or indirectly related to the election, as well as studies and journalistic commentary are prohibited (Art. 69–70). Electoral campaigns are to be financed through private funding and self-financing, with some public aid (Art. 75). Self-financing means any cash or in-kind-finances from a candidate’s, party’s, or list’s own financial resources (Art. 76). Private financing concerns any cash or in-kind from a source other than the candidate, list, or party, by a natural person (Art. 77). There is some public funding for campaigning for each candidate or list. The first half is received before the campaign, the second half a week after the results of the final elections on the condition that there is proof of spending the first half (Art. 78). Candidates are required to repay the public subsidies if they obtained less than 3% of national votes. Lists also must return their public subsidies if they obtain less than 3% of votes cast in their electoral constituency and fail to win seats in the parliament (Art. 78). Campaign financing and in-kind contributions are prohibited from foreign sources, corporations, governments, or foreign non-Tunisian individuals (Art. 80).

### Minor reforms in 2017 and 2019: gender parity, military, and campaign financing

The minor electoral reforms in 2017 (*Loi organique n° 2017‑7 du 14 février* 2017) and 2019 (*Loi organique ° 2019-76 du 30 août *2019)^2^ make smaller amendments pertaining to gender parity in electoral lists, the inclusion to young candidates, as well as disabled candidates, in addition to adjustments in campaign financing of campaigns and penalties. The very short 2019-reform made smaller amendments and specifications to the timeline of re-elections in case of the death of a candidate (Art. 49), and the procedure to appeal electoral results.

While registering for the elections was voluntary in the 2014-electoral law, it was made mandatory in the 2017 law (Art. 7). The duration of the pre-electoral and pre-referendum phases was shortened from three months to only two months, though the main phase for campaigning is still set at 22 days (Art. 50). The amended article 6 lifted previous restrictions on members of paramilitary security personnel and the armed from registering to vote, which were particularly contentious among civilians and even members of the security apparatus (Al-Hilali 2018). Military political participation is, however, restricted to local and municipal elections, banning candidacy in municipal and regional elections, participation in electoral campaigns, partisan meetings, or any activities related to the elections (Art. 49 and 52).

Every candidate or list that achieves more than 3% of votes in an electoral district now receives a public subsidy as a lump sum to reimburse electoral expenses *after* the elections (Art. 78). Fines in case of failure to report or rejection by a court of auditors were raised, while financial penalties were lowered (Art. 98). Clauses concerning gender parity and overall representation were added for municipal and regional councils where candidate lists must alternate between men and women (Art. 49). Lists must include a younger candidate—that is up to 35 years of age—within their top-three candidates, and the rest of the list, as well as a male or female candidate with a physical disability among the top-ten of a list (Art. 49). (Articles 86 and 98).

### Tunisia’s major 2022 reform: doing away with political parties altogether

The 2022 reform (*Decree Law No. 2022-55 of September 15, 2022, amending Organic Law No. 2014-16 of the Year 2014*)^3^ introduces new candidacy requirements in legislative elections, modifies suffrage and voter registry, campaign finance and raises penalties. The reform decreases the size of electoral districts, increases the total number of districts, and assigns one seat to each, wherein candidates are elected in two rounds (Annex A–B of the 2022 Decree Law). Though it does not explicitly address political parties, the major reform it introduces from a closed-list PR-system to a highly candidate-centered system fundamentally uproots the role of political parties or lists in electoral competition altogether.

Candidates now apply individually with their campaign programs and 400 signatures from voters endorsing them, wherein half of the signatures must be from women, a quarter from voters under 35 years of age, and none endorsing any other candidate (Art. 21). Candidates cannot be dual citizens, naturalized citizens without Tunisian parents, or have been charged with any crime, and specific members of the legislature including imams, heads of sports associations, and government employees cannot re-run for a year after their terms (Art. 19–20). In the new system, provisions on gender parity and inclusion of disabled candidates on candidate lists do not apply. Restrictions on voter registration for active-duty military personnel were reintroduced and expanded to any detained citizen, regardless of whether it is due to mental illness or committed crimes (Art. 6).

Campaign subsidies by the state that were mandated in previous reforms were repealed entirely; campaigns must be financed in cash or in-kind exclusively through natural persons and private finances (Art. 75, 81). Funding from foreign sources remains prohibited, but anonymous donations are also prohibited and can be punished by fines, five years of imprisonment, and a ban from running in any election after conviction (Art. 163). If candidates are found to have influenced voters through monetary or in-kind donations, they lose their right to candidacy for life and the right to vote for ten years after conviction (Art. 161). Exploiting other candidates’ personal or locational affiliations and their honor is also punishable by imprisonment for up to five years and a fine (Art. 161).

## Cui bono? The beneficiaries of Tunisia’s electoral reforms

To assess why these reforms failed to mitigate problems with governability and proportionality, it is necessary to outline which political actors stood to gain from which reform first. Table 1 summarizes all major and minor reforms undertaken in Tunisia since 2011 and groups them by reform issue and year of reform in the first column. The reforms are primarily concerned with the financial and administrative pre-requisites of entering electoral competition, but do not address party formation and membership or coalition-building ahead of elections. The “Impact on Electoral Competition”-column summarizes how a particular reform issue impacts electoral competition for specific political actors.

Comparing the reforms across time shows several trends. Overall, all reforms incrementally restrict the electoral system by curtailing specific contenders’ capacity to compete in elections. The major changes undertaken through the 2022-decree uphold this tendency and sideline the role of political parties in electoral competition altogether. The specific prerequisites to compete in elections also increasingly advantage wealthy and older candidates, as well as established older parties with more financial resources available than newer parties. Until 2022, reforms tended to encourage voter turnout for secularist parties, specifically through introducing gender parity voting rights for security and military staff in local and municipal elections—both of which historically secularist domains. This development affirms that when electoral systems subserve incumbents, there will either be little incentive to seek reform or introduce reforms to obstruct new challengers (Núñez et al. 2017; Remmer 2008; Siavelis 1997).

### Campaign and pre-campaign periods: established candidates and parties gain advantage

Tunisia’s very short electoral campaign and pre-electoral campaign period leave little space to engage with voters, other parties, and future coalition partners in general. That constricts the range of issues that can realistically be addressed within a campaign period in general. Especially in the context of a young democratic polity with chronically low voter turnout and a high level of distrust towards political parties, attracting a voter base and thus institutionalizing a political party naturally takes more time. Older political parties, such as Ennahda or Bourguibist parties, that have comparatively clear political stances and a voter base do not need to rely as strongly on campaigning to gain a competitive advantage. Newly established parties on the other hand need the campaign period to build a voter base from scratch while also competing against other parties. As volatility and factionalism afflicted smaller parties on the secularist side of the political spectrum, the short campaign period affects competition for these parties more than for institutionalized parties like Ennahda.

### Campaign finance and penalties: burden and leverage on new contenders and opponents

Succeeding in electoral competition requires financial, personnel, and in-kind resources before the actual campaign period. The gradual limitation on campaign finance and subsidies from the state restricts political contenders because campaign finance is increasingly dependent on a candidates’ personal finances as well as other individuals’ campaign funding. The approach laid out in the 2014 electoral law with one installment before and after an election gave some subsidies to new parties, candidates, or lists, partially lifting financial burdens. From 2017 on however, parties received their public subsidies in a lump sum after the elections and only if they won either 3% of votes or a seat in the parliament. Refunding expenses after the election also presupposes that candidates, lists, or political parties already have enough personal financial resources ahead of the election to finance their campaigns. The ban on campaign funding from abroad, corporations, or foreign governments, but also anonymous financial and in-kind donations reinforce the reliance on personal financial resources.

This disadvantages younger candidates who are more likely afflicted by Tunisia’s enduring socio-economic grievances since the Arab Spring and cannot diversify their funding sources if they do not have financial resources at their disposal already. Consequently, older, and more established candidates, parties and lists gain a substantive competitive advantage. Wealthier candidates can run more easily and found personalist political parties, as it has been the case with the Free Patriotic Union, which was launched and funded by a wealthy businessman (Ryan 2011). The strong dependence of campaigns on the financial resources of specific individuals or interest groups fuels personalist tendencies in electoral systems in addition to the clientelist relationship between already dominant parties and the state (Fisher and Eisenstadt 2016). The cancellation of subsidies through the 2022-reform further entrenches the structural bias against less wealthy candidates.

Through increasingly high penalties such as imprisonment and bans on candidacy for years, campaign finance can be leveraged against both new and established candidates, but also voters who lose their right to vote by principle if accused and convicted of any crime. A case in point are recurrent accusations and investigates of Ennahda-members for alleged funding from the Gulf and affiliation with the Muslim Brotherhood (Al-Monitor 2021).

### Suffrage for military and security personnel: impact offset through general conscription

Endowing security and military staff with voting rights in the 2017-reform seemed at odds with the constitutionally mandated political impartiality of the army and its own history. It is therefore unsurprising that this reform was not received well by the public until it was repealed in 2022. In this case, however, military suffrage served secular political actors rather than the military institution itself. Ben Ali came to power through a bloodless coup d’état against his predecessor Habib Bourguiba in 1987, and both undertook active measures to ban the military from Tunisia’s political arena and any political activity (Bou Nassif 2015). Tunisia has general conscription, and military recruits often share the socio-economic grievances of civilians (Lutterbeck 2013).

From Bourguiba to Ben Ali, the regime’s security apparatus retained its base within the police corps, which grew unpopular through its heavy-handed repression of the Islamic Tendency Movement, Ennahda’s early precursor (Sadiki 2002). The police and armed forces were used to repress Ennahda and other Islamist movements as common unifying enemy throughout Ben Ali’s rule and were traditionally secular domains. The military’s defection from Ben Ali during the Arab Spring cemented the military’s popularity among civilians (Kirkpatrick 2011). Anti-establishment attitudes and general dissatisfaction with new democratic institutions continued to drive military support (Albrecht et al. 2021). Nidaa Tounes supporters favoured a role expansion of the military, and supporters of the Ennahda Party did not actively oppose an expansion of military role either (Albrecht et al. 2021, p. 12). Though military suffrage was highly contentious and gave a slight competitive advantage to secularist candidates and parties, its impact is easily offset through general conscription.

### Gender parity and inclusion: effects can go either way

How clauses on parity and inclusion impact a candidates’ or parties’ capacity to compete varies across location and social class, and can go either way. Secular political parties stand to gain from gender parity because women rallied around matters concerning development and modernization (Al-Ali 2003). Until the ouster of Ben Ali, women were a traditionally secular domain in Tunisian electoral politics (Grami 2018). The first family code law enacted by Bourguiba guaranteed women access to education and employment, which won widespread support among women (Arfaoui and Tchaïcha 2014). Urban and educated women were likewise supportive of Ben Ali due to his commitment to the most progressive family and gender law in the Middle East, as women saw their rights jeopardized by Islamists (Angrist 2013).

The fear over what Islamist rule by Ennahda and other Islamist parties may entail for women’s rights, continued to push many female voters towards secular parties (Arfaoui and Tchaïcha 2014, pp. 145–146). Likewise, Nidaa Tounes attracted many women fearing their rights and liberties may be in jeopardy, but disappointed when only one woman was named at the top of the list for the 2014 elections (L. Labidi 2014). As voters punished Nidaa Tounes for collaborating with the Ennahda party, Ennahda tried to expand its position by promoting moderate Islam to secure women’s support (L. Labidi 2014, p. 9). As secular women’s movements were typically championed by educated middle-class women, Islamist women’s movements were pushed by women in the lower- and lower-middle classes (Debuysere 2016). Islamist women’s activism is not only concentrated in working-class neighbourhoods, but also in rural areas and Tunisia’s poorer south which had been neglected by the urban-concentrated secular women’s movements (Debuysere 2016, p. 234).

Wherever secularist political actors do not cater to women from the lower and lower middle class, Ennahda could gain an advantage. Forced consensus-building between secularists and Islamists in the ‘Government of National Unity’ after the 2016 Carthage-Agreement deterred secular voters (Dihstelhoff and Sold 2016). As a rule though, the more requirements and quotas are introduced, the higher the burden on new and small contenders with limited resources. How the expiration of parity and inclusion clauses plays out in the long term through the new candidate-centred electoral system is still uncertain at the time of writing.

## Did reforms miss the point of proportionality and governability?

There is always some form of trade-off between proportionality and governability. Parties ideally transpose a political system’s underlying societal structure, and therefore voters’ demands into a political system. Fewer parties technically also facilitate governability. To a certain extent, fragmentation fosters proportionality because it makes way for smaller actors to push electoral changes and represent niche interests. Yet, Tunisians were seemingly getting the short end of the stick for both. So, why have these reforms failed to improve both governability and proportionality?

Though Tunisia’s unstable party landscape is beyond the scope of this analysis, electoral systems cannot be assessed without also considering some features of a party system (Sartori 1994). Volatility and fragmentation of the party system were powerful drivers of Tunisia’s electoral reforms. Increasing proportionality through fragmentation presupposes that political parties fulfill their core function to adequately represent voters. Yet, fragmentation within Tunisia’s landscape is not due to smaller parties representing niche interests, but rather due to intra-party factionalism and short-lived coalition building between elections.

Tunisia’s proportionality problem is rooted in the quality of representation through existing political parties. Against the volatile secularist landscape, Ennahda became a political party that holds both coalition *and *blackmailing potential in Sartori’s (1999) terminology. Though its overall share in parliamentary seats declined, governance without Ennahda was and is difficult; secularist parties either needed to enter coalitions with Ennahda, steamrolling ideological differences and alienating voters on both sides to the detriment of all parties involved after the Troika-government. Parties also had to form very broad and short-lived coalitions among them to counter Ennahda’s relative hegemony, which forced smaller and wing parties into a centrist stance (Green-Pedersen 2004).

Fewer parties within the legislature do not improve governability when the parties themselves cannot fulfill their core functions due to salient intra-party factionalism, weak institutionalization, and a short half-life between cabinets and elections. Tunisia’s electoral laws contributed little to diffuse these issues and could even worsen the situation if electoral laws continue to skew the playing field in favor of wealthier parties and candidates. The 2022 reform not only sidesteps the issue—it all but eliminates the role of political parties.

## Conclusion

Achieving governability through consensus and collaboration between few parties lead to the political crisis ahead of the 2014 election, but the increasing number of political parties set the stage for the 2021 crisis. Restricting the parties should have improved governability in theory. Until 2022, Tunisia’s electoral system was designed to constrain the number of parties, and the reform of 2022 de-emphasized their importance in the electoral system altogether. But without measures to improve the quality of representation by political parties, artificial constraints on who can compete in elections cannot improve proportionality. Both major and minor reforms failed to address the quality of political representation.

Tunisia’s trajectory since the Arab Spring stood out because of its initial democratization and high attention to both major and minor reforms by democracy observers. But the challenge of crafting effective electoral legislation, curbing fragmentation in the electoral arena, and ensuring governability extends well beyond it. Studying Tunisia’s electoral reforms longitudinally points to several avenues for future research.

The role of minor reforms has been largely overlooked in previous literature. Likewise, research on how campaign finance can be strategically leveraged in electoral competition against specific parties or candidates is scant. Incumbents can use legislation to entrench their strategic interests into the fabric of political systems. Mapping the trajectory of both major and minor reforms in this paper has shown that these changes can be undertaken incrementally over time without upending political systems entirely. By focusing on singular and major electoral reforms only, studies lose out on many other factors that can chip away at the proportionality of electoral systems. Even seemingly minor reforms can have salient impacts on electoral competition over time.

Troubleshooting Tunisia’s electoral system can give crucial insight for other MENA-countries where regime change stalled over disputes in electoral legislation, especially in the forerun of the first post-transition elections and constitutional reforms. Dysfunctional party systems and the inability to transpose existing cleavages into a civilian and functioning political arena is a recurring issue across the MENA-region where parties are consistently among the least trusted political institutions (Jamal et al. 2020). Understanding how this feeds into the proportionality-governability tradeoff can generate valuable insights for the design of electoral systems after regime transitions and in the course of peace processes. Relatedly, studying Tunisia’s electoral system in detail emphasizes that definitions of proportionality should not be restricted to the number of political parties in a political system. A high number of political parties is not a reliable indicator of proportionality if parties are dysfunctional and weakly institutionalized to begin with. Electoral systems can offset or reinforce the effectiveness of political parties as political vehicles in electoral competition. But regulating the electoral process alone cannot fix a dysfunctional political arena.

## Footnotes

1 Original document in French. Translations by the author.

2 Both organic laws originally in French. Translations by the author.

3 Presidential decree published in Arabic. Translations by the author.

## References

[CR55] 2014. Tunisia’s constitution of 2014. https://www.constituteproject.org/constitution/Tunisia_2014.pdf. Accessed 20 Dec 2022.

[CR2] AFP. 2021. Tunisian president takeover sparks fears for freedoms. https://www.france24.com/en/live-news/20210822-tunisian-president-takeover-sparks-fears-for-freedoms (Created 22 Aug 2021). Accessed 20 Dec 2022.

[CR3] Al-Ali, Nadje. 2003. Gender and civil society in the Middle East. *International Feminist Journal of Politics* 5(2):216–232.

[CR4] Al-Hilali, Amel. 2018. Tunisia’s military votes for the first time in 62 years.* Al-Monitor*. https://www.al-monitor.com/originals/2018/05/tunisian-military-staff-cast-ballots-first-time-elections.html (Created 11 May 2018). Accessed 20 Dec 2022.

[CR7] Al-Monitor. 2021. Leading tunisian Islamist party under investigation for alleged foreign funding.* Al-Monitor*. https://www.al-monitor.com/originals/2021/07/leading-tunisian-islamist-party-under-investigation-alleged-foreign-funding (Created 28 July 2021). Accessed 20 Dec 2022.

[CR1] Albrecht, Holger, Michael Bufano, and Kevin Koehler. 2021. Role model or role expansion? Popular perceptions of the military in Tunisia. *Political Research Quarterly*10.1177/10659129211001451.

[CR5] Ali, Hager, Salah Ben Hammou, and Jonathan Powell. 2022. Between coups and election: Constitutional engineering and military entrenchment in Sudan. *Africa Spectrum* 57(2):1–13. 10.1177/00020397221136581.

[CR6] Aliriza, Fadil. 2022. Saied’s new rules for Tunisia’s elections. https://www.mei.edu/publications/saieds-new-rules-tunisias-elections. Accessed 20 Dec 2022.

[CR8] Amara, Tarek. 2021. Protests across Tunisia target Ennahda party over political crisis.* Reuters*. https://www.reuters.com/world/africa/protests-across-tunisia-covid-19-surges-economy-suffers-2021-07-25/. Accessed 20 Dec 2022.

[CR9] Angrist, Michele. 2013. Understanding the success of mass civic protest in Tunisia. *The Middle East Journal* 67(4):547–564.

[CR10] Arfaoui, Khedija, and Jane Tchaïcha. 2014. Governance, women, and the new Tunisia. *Politics and Religion Journal* 8(1):135–164.

[CR11] Battera, Frederico, and Ieraci Giuseppe. 2019. Party system and political struggle in Tunisia. Cleavages and electoral competition after the transition to democracy. *Polyarchie/Polyarchies* 2(1):4–44.

[CR12] Birch, Sarah. 2011. *Electoral malpractice*. Oxford: Oxford University Press.

[CR13] Bou Nassif, Hicham. 2015. A military besieged: The armed forces, the police, and the party in Bin ’Ali’s Tunisia, 1987–2011. *International Journal of Middle East Studies* 47(1):65–87.

[CR14] Boubekeur, Amel. 2015. Islamists, secularists and old regime elites in Tunisia: bargained competition. *Mediterranean Politics* 21(1):107–127. 10.1080/13629395.2015.1081449.

[CR15] Boulby, Marion. 1988. The Islamic challenge: Tunisia since independence. *Third World Quarterly* 10(2):590–614.

[CR16] Carey, John, and Andrew Reynolds. 2011. The impact of election systems. *Journal of Democracy* 22(4):36–47.

[CR17] Colomer, Josep. 2004. The strategy and history of electoral system choice. In *The handbook of electoral system choice*, ed. J.M. Colomer, 3–78. Houndsmills, Basingstoke, Hampshire, New York: Palgrave.

[CR18] Debuysere, Loes. 2016. Tunisian women at the crossroads: Antagonism and agonism between secular and Islamist women’s rights movements in Tunisia. *Mediterranean Politics* 21(2):226–245.

[CR19] Dihstelhoff, Julius, and Katrin Sold. 2016. The Carthage agreement under scrutiny.* Carnegie endowment for international peace*. https://carnegieendowment.org/sada/66283. Accessed 20 Dec 2022.

[CR20] Fisher, Justin, and Todd A. Eisenstadt. 2016. Introduction: comparative party finance. *Party Politics* 10(6):619–626.

[CR21] France 24. 2022. Référendum en Tunisie : la nouvelle Constitution adoptée malgré une forte abstention. Tunis: France24. https://www.france24.com/fr/afrique/20220726-r%C3%A9f%C3%A9rendum-en-tunisie-la-nouvelle-constitution-adopt%C3%A9e-%C3%A0-plus-de-94 (Created 27 July 2022). Accessed 20 Dec 2022.

[CR22] Gobe, Eric. 2017. De la dialectique du “local” et du “national” dans les lois électorales tunisiennes ou comment représenter le “peuple” dans la Tunisie post-Ben Ali. *L’Année du Maghreb* 16(2017):153–170. 10.4000/anneemaghreb.3023.

[CR23] Grami, Amel. 2018. Women, feminism and politics in post-revolution Tunisia: Framings, accountability and agency on shifting grounds. *Feminist Dissent* 3:23–56.

[CR24] Green-Pedersen, Christoffer. 2004. Center parties, party competition, and the implosion of party systems: A study of centripetal tendencies in multiparty systems. *Political Studies* 52:324–341.

[CR27] Hamid, Shadi. 2014. Political party development before and after the Arab spring. In *Beyond the Arab spring: the evolving ruling bargain in the middle Eas*, ed. Mehran Kamrava, 131–150. Oxford: Oxford University Press.

[CR25] Jamal, Amaney, Michael Robbins, and Salma Al-Shami. 2020. Youth in MENA: Findings from the fifth wave of the arab barometer. https://www.arabbarometer.org/publication/youth-in-mena-findings-from-the-fifth-wave-of-the-arab-barometer/. Accessed 20 Dec 2022.

[CR26] Jawa, Rana. 2021. Tunisia’s PM sacked after violent Covid protests.* BBC News*. https://www.bbc.com/news/world-africa-57958555 (Created 26 Nov 2021). Accessed 20 Dec 2022.

[CR28] Katz, Richard S. 2005. Why are there so many (or so few) electoral reforms? In *The politics of electoral systems*, ed. M. Gallagher, P. Mitchell, 57–76. Oxford: Oxford University Press.

[CR29] Kirkpatrick, David. 2011. Chief of tunisian army pledges his support for ‘the revolution’.* The New York Times*. https://www.nytimes.com/2011/01/25/world/africa/25tunis.html (Created 24 Jan 2011). Accessed 20 Dec 2022.

[CR30] Labidi, Kamel. 2019. The time is ripe for comprehensive reform in Tunisia.* Open Democracy*. https://www.opendemocracy.net/en/north-africa-west-asia/time-ripe-comprehensive-reform-tunisia/. Accessed 20 Dec 2022.

[CR31] Labidi, Lilia. 2014. Electoral practice of Tunisian women in the context of a democratic transition. https://www.wilsoncenter.org/sites/default/files/media/documents/publication/electoral_practice_of_tunisian_women_in_the_context_of_a_democratic_transition.pdf. Accessed 20 Dec 2022.

[CR32] Lijphart, Arend. 1990. Consequences of electoral laws, 1945–85. *The American Political Science Review* 84(2):481–496.

[CR33] Loi organique n° 2014-16 du 26 mai 2014, relative aux élections et référendums. 2014. Journal Officiel de la République Tunisienne N°42(27.05.2014): 1310–1331. https://legislation-securite.tn/law/44286. Accessed 20 Dec 2022.

[CR34] Loi organique n° 2017‑7 du 14 février 2017, modifiant et complétant la loi organique n° 2014-16 du 26 mai 2014 relative aux élections et référendums. http://www.isie.tn/wp-content/uploads/2018/01/Loi-Organique-n%C2%B02017-7.pdf. Accessed 20 Dec 2022.

[CR35] Loi organique n° 2019-76 du 30 août 2019, modifiant et complétant la loi organique n° 2014-16 du 26 mai 2014, relative aux élections et référendums. https://legislation-securite.tn/law/42116. Accessed 20 Dec 2022.

[CR36] Lutterbeck, Derek. 2013. Arab uprisings, armed forces, and civil—Military relations. *Armed Forces & Society* 39(1):28–52.

[CR37] Mainwaring, Scott. 1991. Politicians, parties, and electoral systems: Brazil in comparative perspective. *Comparative Politics* 24(1):21–43.

[CR38] McElwain, Kenneth. 2008. Manipulating electoral rules to manufacture single-party dominance. *American Journal of Political Science* 52(1):32–47.

[CR39] Núñez, Lidia, Pablo Simón, and Jean-Benoit Pilet. 2017. Electoral volatility and the dynamics of electoral reform. *West European Politics* 40(2):378–401.

[CR40] Patkós, Veronika, and Árpad Stump. 2022. Do electoral reforms tend to favor the incumbents? A quantitative analysis. *Acta Politica*10.1057/s41269-022-00237-8.

[CR41] Presidential Decree Law No. 2022-55 of September 15, 2022, amending Organic Law No. 2014-16 of the Year 2014 Related to Elections, Referenda and their Conduct. 2022. DECAF Tunisie (orig. Version in Arabic). https://www.businessnews.com.tn/bnpdf/JournalArabe1022022-1.pdf?fbclid=IwAR0-hryReA00qUgWid3480SRRDPdGKBUj_jjuorIZruYH8V9Si23tLX-GGA. Accessed 20 Dec 2022.

[CR42] Rahat, Gideon. 2014. The politics of electoral reform: the state of research. *Journal of Elections, Public Opinion, and Parties* 21(4):523–543.

[CR43] Remmer, Karen L. 2008. The politics of institutional change. *Party Politics* 14(1):5–30.

[CR44] Renwick, Alan. 2010. *The politics of electoral reform. Changing the rules of democracy*. Cambridge: Cambridge University Press.

[CR45] Reuters. 2021. Tunisia says health care system collapsing due to COVID-19.* Reuters*. https://www.reuters.com/business/healthcare-pharmaceuticals/tunisia-says-health-care-system-collapsing-due-covid-19-2021-07-08/ (Created 8 July 2021). Accessed 20 Dec 2022.

[CR46] Ryan, Yasmine. 2011. Tunisian newcomer spends big on campaign.* Al Jazeera*. https://www.aljazeera.com/features/2011/10/21/tunisian-newcomer-spends-big-on-campaign (Created 21 Oct 2011). Accessed 20 Dec 2022.

[CR47] Sadiki, Larbi. 2002. Bin Ali’s Tunisia: democracy by non-democratic means. *British Journal of Middle Eastern Studies* 29(1):57–78.

[CR48] Sadiki, Larbi. 2011. Civic Islamism: the Brotherhood and Ennahdha.* Aljazeera*. https://www.aljazeera.com/opinions/2011/11/15/civic-islamism-the-brotherhood-and-ennahdha (Created 15 Nov 2011). Accessed 20 Dec 2022.

[CR58] Sarah, Yerkes, and Mohammad Al-Mailam. 2022. Tunisia’s new electoral law is another blow to its democratic progress. *Carnegie endowment for international peace*(11.10.2022): 1–5. https://carnegieendowment.org/2022/10/11/tunisia-s-new-electoral-law-is-another-blow-to-its-democratic-progress-pub-88127. Accessed 20 Dec 2022.

[CR49] Sartori, Giovanni. 1994. *Comparative constitutional engineering. An inquiry into structures, incentives and outcomes*. Houndsmills, Basingstoke, Hampshire, London: Palgrave Macmillan.

[CR50] Sartori, Giovanni. 1999. The party-effects of electoral systems. *Israel Affairs* 6(2):13–28.

[CR51] Schedler, Andreas. 2006. *Electoral authoritarianism: the dynamics of unfree competition*. Boulder: Lynne Rienner.

[CR53] Shugart, Matthew. 2005. Comparative electoral systems research: the maturation of a field and new challenges ahead. In *The politics of electoral systems*, ed. Michael Gallagher, Paul Mitchell, 25–56. Oxford: Oxford University Press.

[CR52] Siavelis, Peter. 1997. Continuity and change in the Chilean party system. On the transformational effects of electoral reform. *Comparative political studies* 30(6):651–674.

[CR54] Tavana, Daniel. 2018. *The evolution of the Kuwaiti ‘opposition’: electoral politics after the Arab spring*. Rice University’s Baker Institute for public Policy policy brief 08.07.2018., 1–4.

[CR56] Yerkes, Sarah. 2019. Tunisia’s elections explained. The world’s youngest democracy votes.* Carnegie endowment for international peace*. https://carnegieendowment.org/publications/interactive/tunisian-elections-2019. Accessed 20 Dec 2022.

[CR57] Yerkes, Sarah, and Zeineb Ben Yahmed. 2019. Tunisia’s political system: from stagnation to competition. *Carnegie endowment for international peace*(28.03.2019), 1–16. https://carnegieendowment.org/2019/03/28/tunisia-s-political-system-from-stagnation-to-competition-pub-78717. Accessed 20 Dec 2022.

